# Supplementary figures and images for: Identification of potentially effective drugs for metabolic dysfunction-associated steatotic liver disease against liver cirrhosis: In-silico drug repositioning-based retrospective cohort study
Source: PLoS One. 2025 Jun 4;20(6):e0323880. doi: 10.1371/journal.pone.0323880 (PMC12136429; doi:10.1371/journal.pone.0323880)

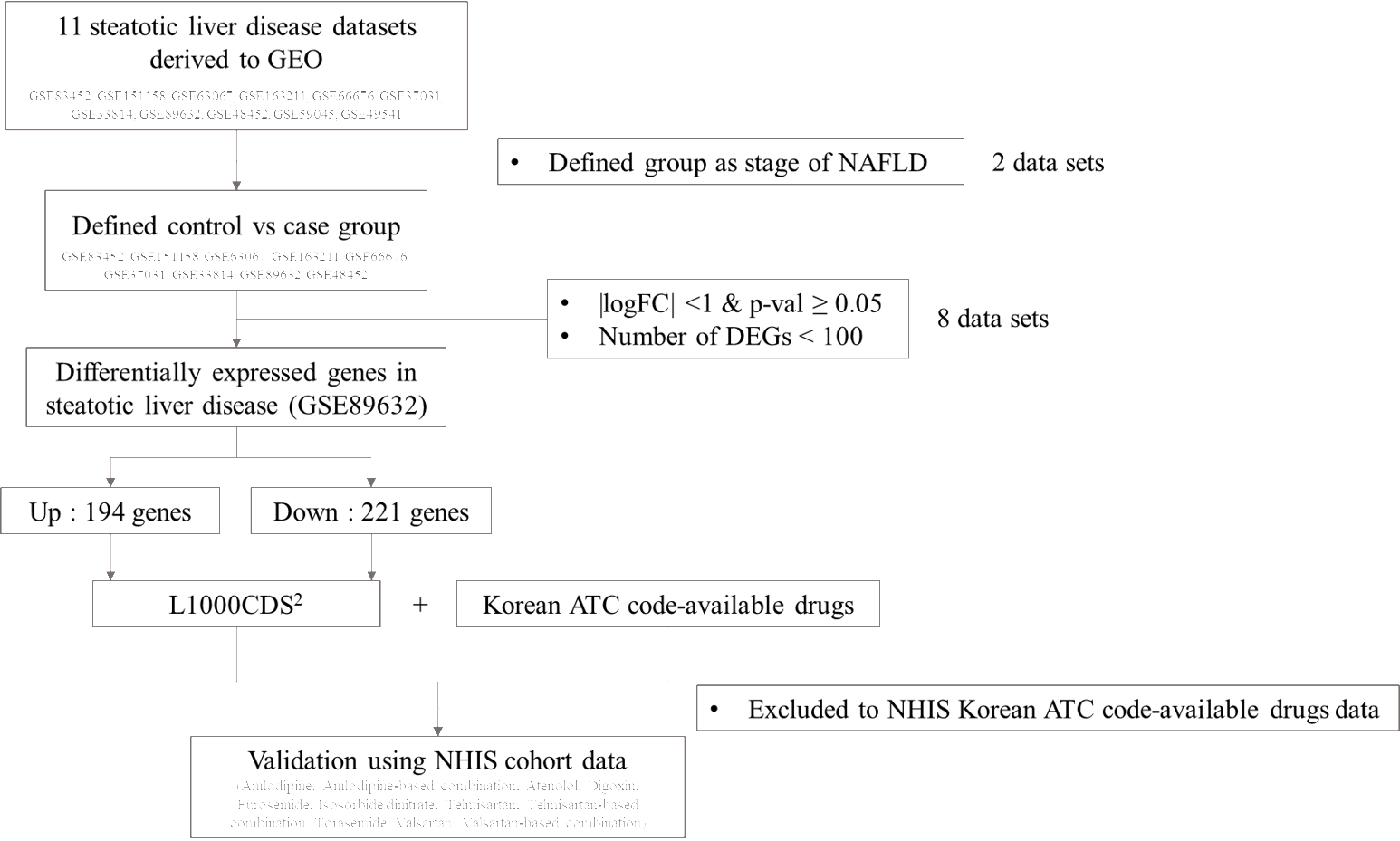

Supplement: S1 Fig — (TIF) [file pone.0323880.s001.tif]

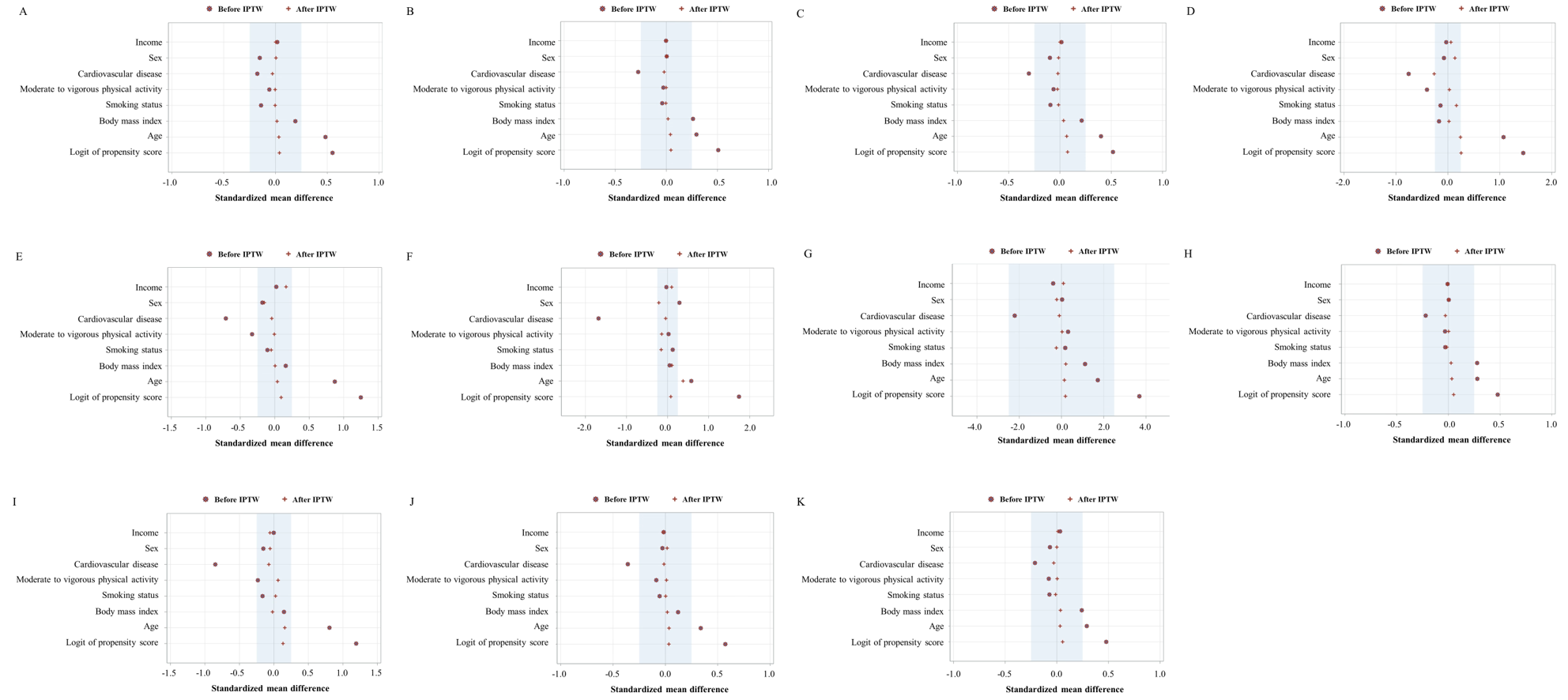

Supplement: S2 Fig — Treated group indicates at least 30 days of cumulative days of the specified drug use. (A) Amlodipine. (B) Amlodipine-based combination. (C) Atenolol. (D) Digoxin. (E) Furosemide. (F) Isosorbide dinitrate. (G) Telmisartan. (H) Telmisartan-based combination. (I) Torasemide. (J) Valsartan. (K) Valsartan-based combination. (TIF) [file pone.0323880.s002.tif]

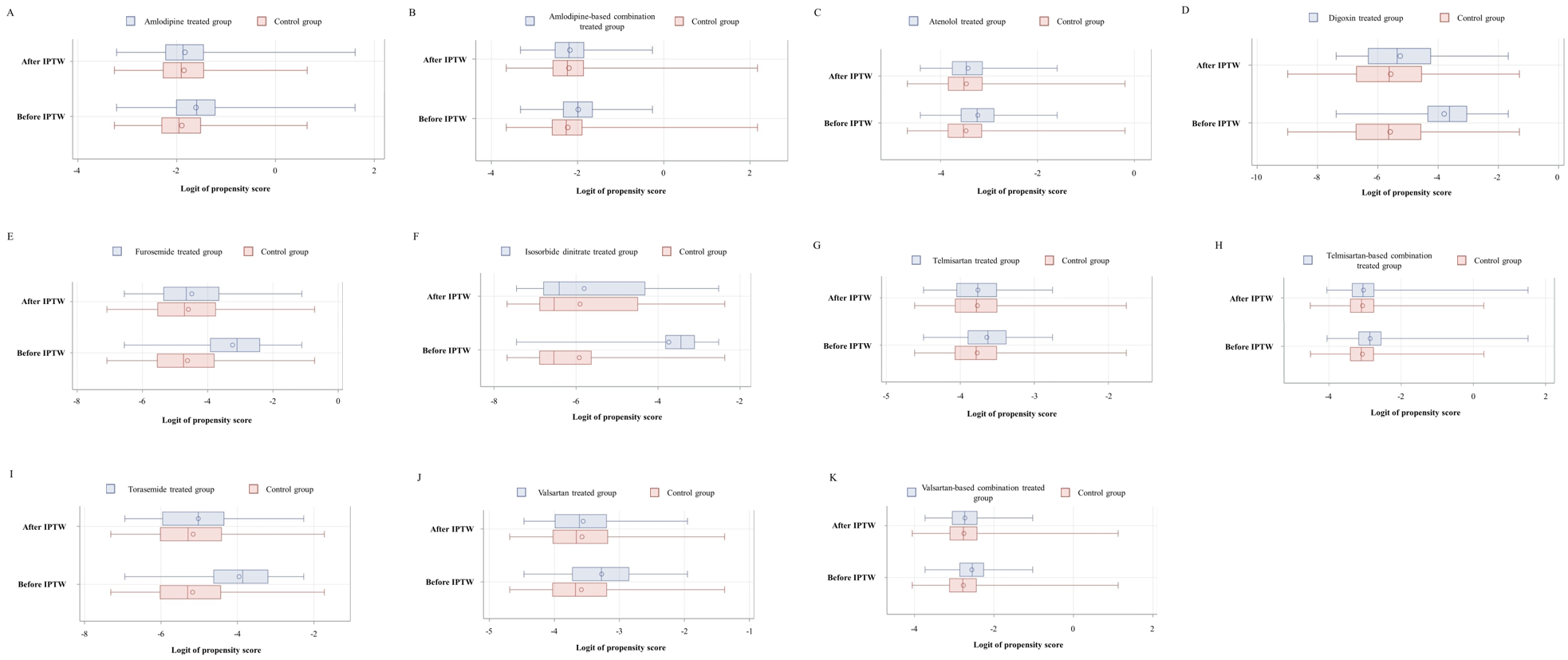

Supplement: S3 Fig — (A) Amlodipine. (B) Amlodipine-based combination. (C) Atenolol. (D) Digoxin. (E) Furosemide. (F) Isosorbide dinitrate. (G) Telmisartan. (H) Telmisartan-based combination. (I) Torasemide. (J) Valsartan. (K) Valsartan-based combination. (TIF) [file pone.0323880.s003.tif]

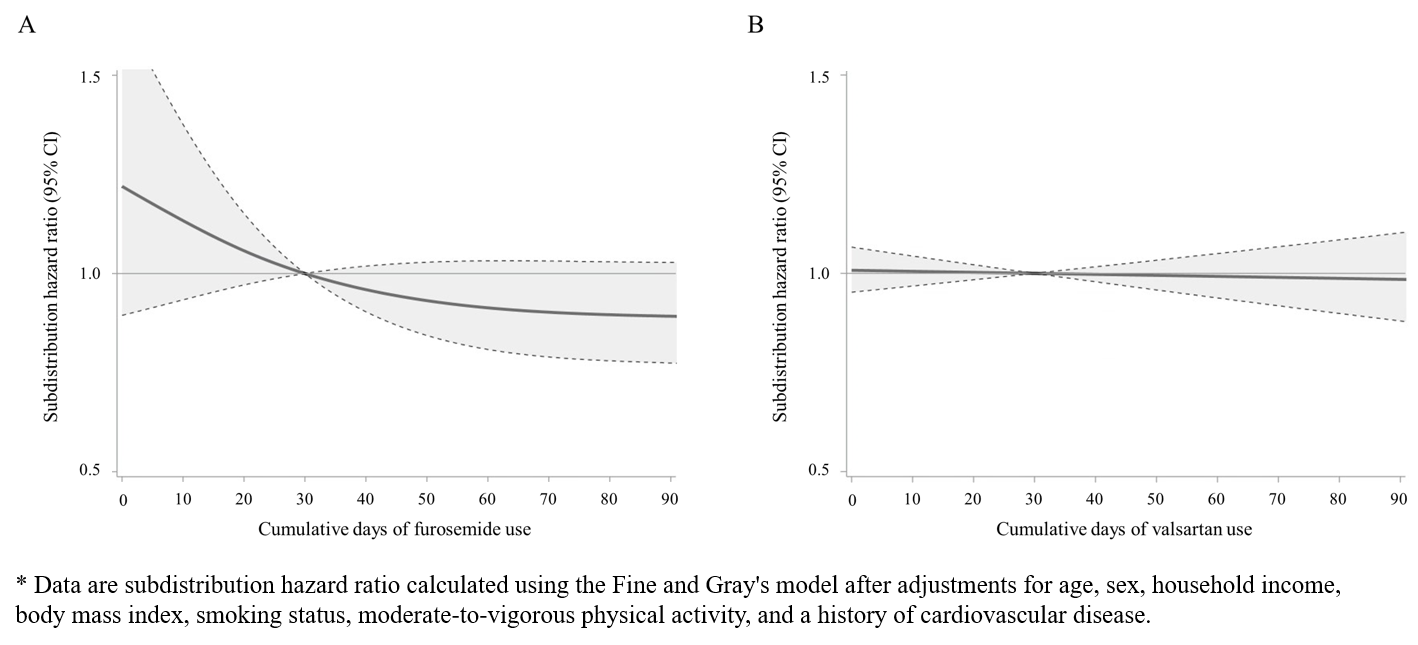

Supplement: S4 Fig — (A) Furosemide. (B) Valsartan. (TIF) [file pone.0323880.s004.tif]

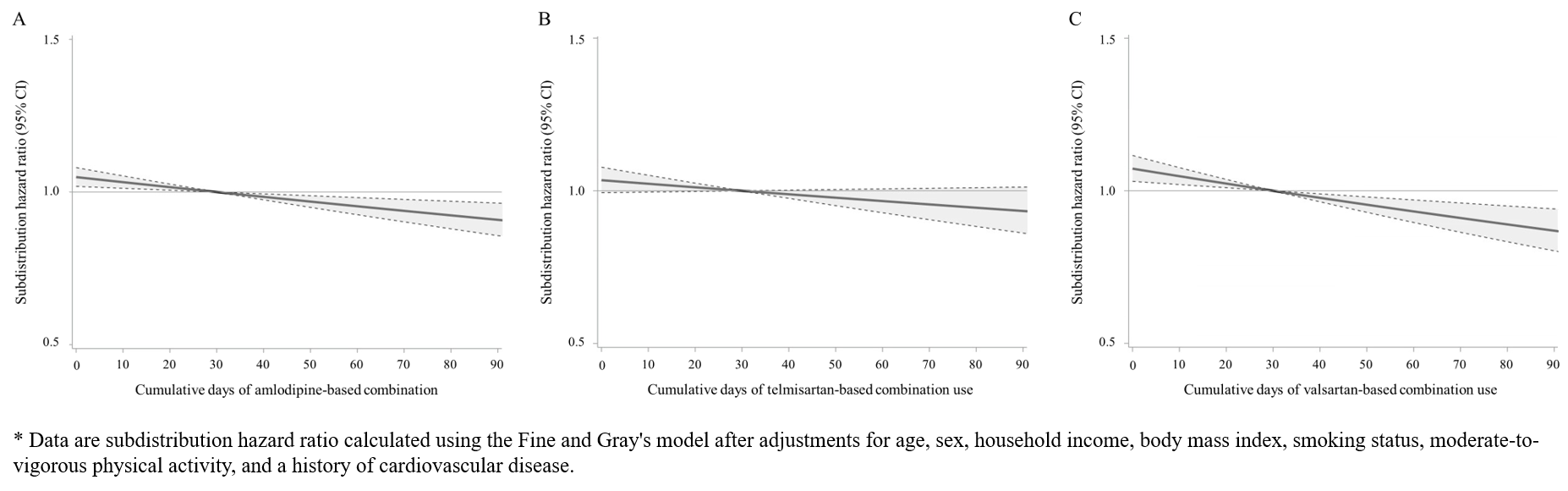

Supplement: S5 Fig — (A) Amlodipine-based combination. (B) Telmisartan-based combination. (C) Valsartan-based combination. (TIF) [file pone.0323880.s005.tif]

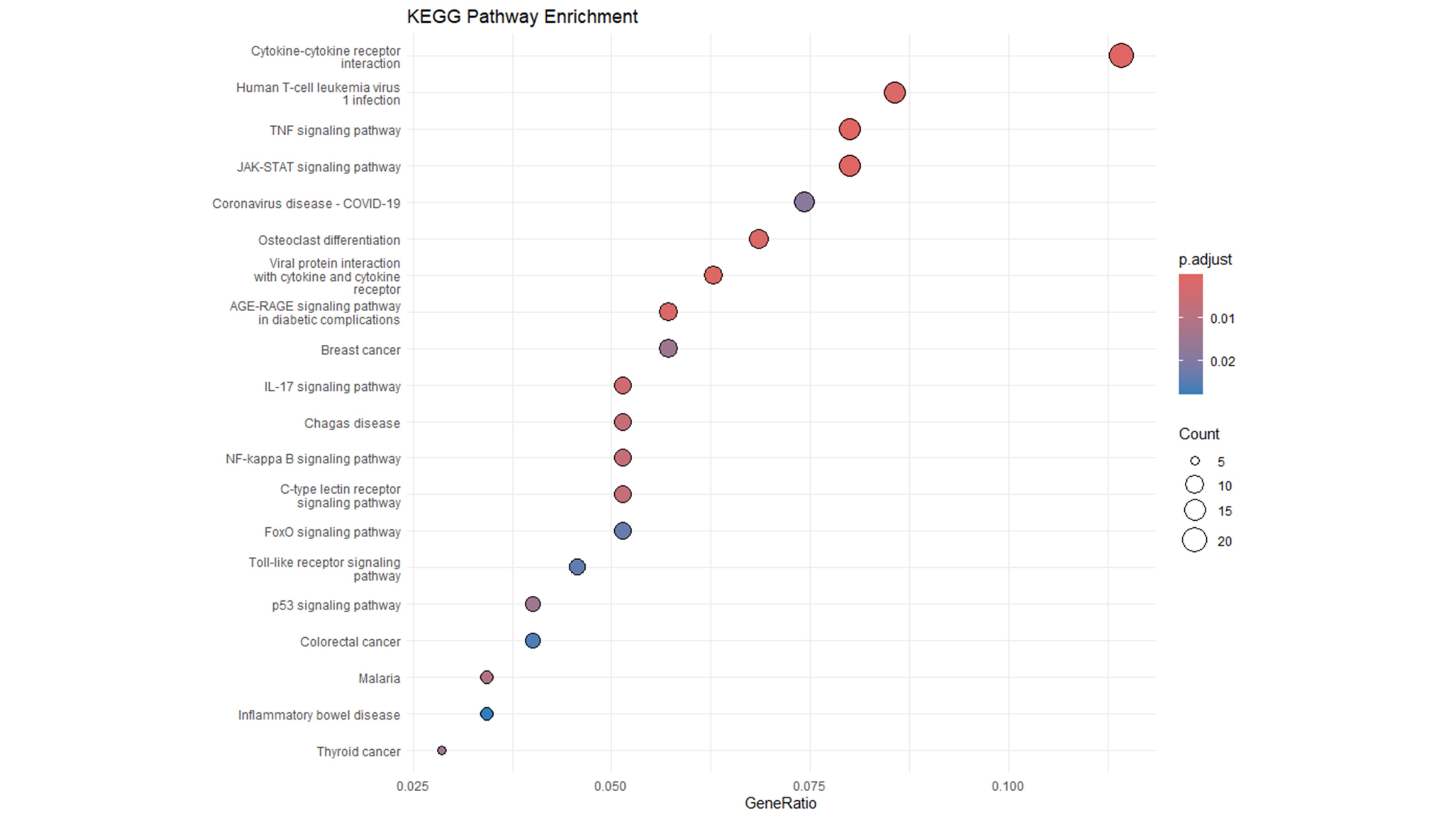

Supplement: S6 Fig — Dot plot showing the enriched KEGG pathways based on the input gene set. The y-axis lists the names of the significantly enriched pathways, while the x-axis represents the gene ratio. Dot size corresponds to the number of genes mapped to each pathway, and dot color indicates the adjusted p-value, with red representing more significant enrichment. (TIF) [file pone.0323880.s006.tif]

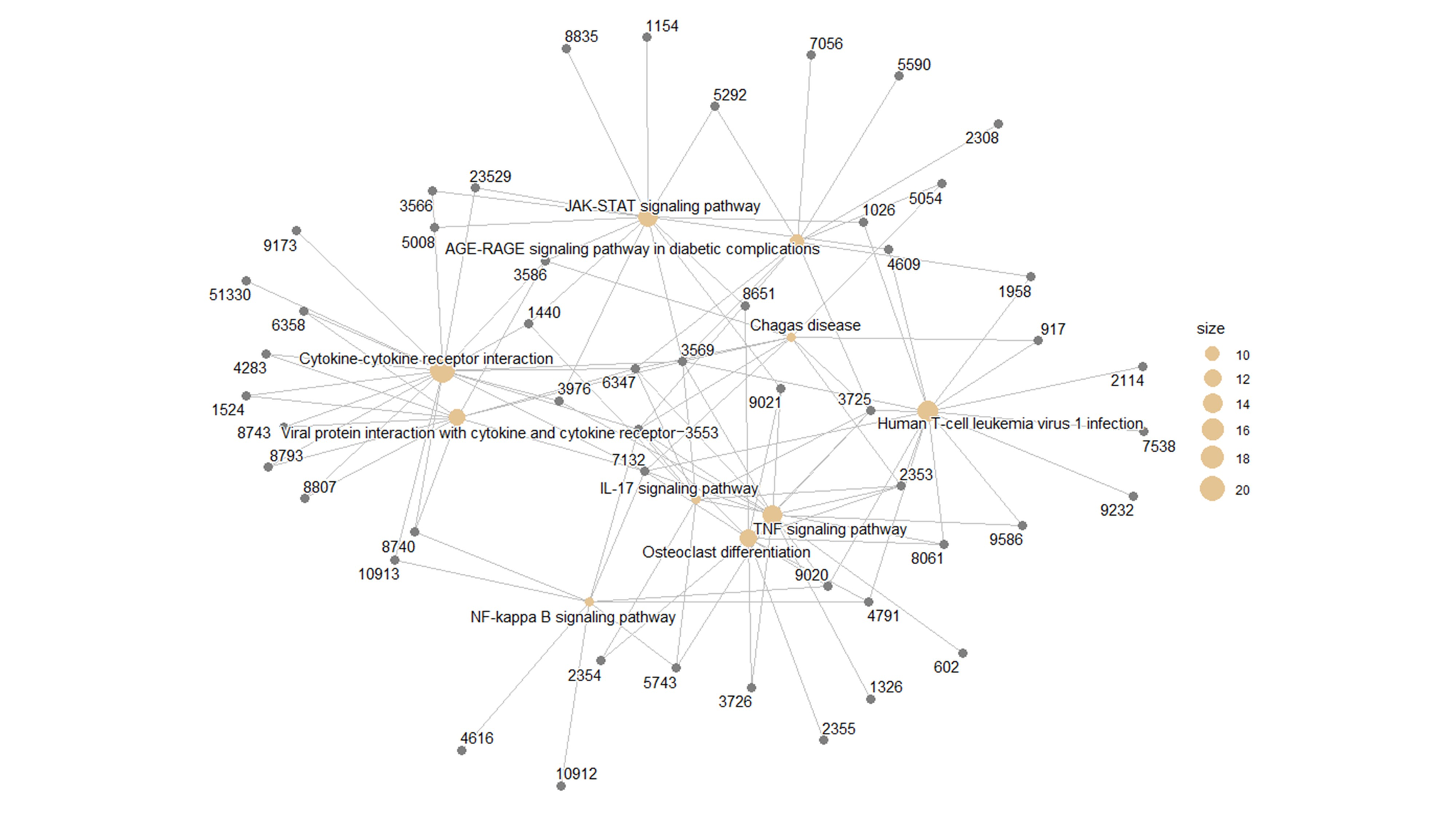

Supplement: S7 Fig — This network plot visualizes the relationships between significantly enriched KEGG pathways and their associated genes. Orange nodes represent KEGG pathways, and gray nodes represent individual genes. Edges indicate that a gene is involved in the connected pathway. The size of each pathway node reflects the number of genes it contains. (TIF) [file pone.0323880.s007.tif]
