# Supplementary material for: Identification of potentially effective drugs for metabolic dysfunction-associated steatotic liver disease against liver cirrhosis: In-silico drug repositioning-based retrospective cohort study
Source: PLoS One. 2025 Jun 4;20(6):e0323880. doi: 10.1371/journal.pone.0323880 (PMC12136429; doi:10.1371/journal.pone.0323880)
Supplement: S1 Table — (DOCX) [file pone.0323880.s008.docx]

**S1 Table. Minimally adjusted subdistribution hazard ratios for drug use against the risk of liver cirrhosis**

| **Drugs** | **SHR (95% CI)** | ***P* value** |
| --- | --- | --- |
| Amlodipine | 1.00 (0.89-1.12) | 0.971 |
| Amlodipine-based combination | 1.25 (1.12-1.41) | <0.001 |
| Atenolol | 0.83 (0.73-0.94) | 0.003 |
| Digoxin | 1.30 (1.16-1.46) | <0.001 |
| Furosemide | 0.99 (0.88-1.12) | 0.927 |
| Isosorbide dinitrate | 0.87 (0.77-0.98) | 0.025 |
| Telmisartan | 0.92 (0.82-1.04) | 0.197 |
| Telmisartan-based combination | 0.94 (0.83-1.06) | 0.295 |
| Torasemide | 1.44 (1.29-1.61) | <0.001 |
| Valsartan | 0.52 (0.45-0.60) | <0.001 |
| Valsartan-based combination | 1.24 (1.11-1.39) | <0.001 |

Data are subdistribution hazard ratio calculated using the Fine and Gray’s model after adjustments for age, sex, household income, body mass index, smoking status, moderate-to-vigorous physical activity, and a history of cardiovascular disease. Wash-out period was defined as the period from the start of the follow-up investigation until specified time.
