# Supplementary material for: Identification of potentially effective drugs for metabolic dysfunction-associated steatotic liver disease against liver cirrhosis: In-silico drug repositioning-based retrospective cohort study
Source: PLoS One. 2025 Jun 4;20(6):e0323880. doi: 10.1371/journal.pone.0323880 (PMC12136429; doi:10.1371/journal.pone.0323880)
Supplement: S2 Table — (DOCX) [file pone.0323880.s009.docx]

**S2 Table. Sensitivity analysis on the risk of liver cirrhosis according to the cumulative days of drug use after excluding events that occurred within specified periods.**

| **Wash-out periods** | **<30 days** | **≥30 days** | ***P* value** |
| --- | --- | --- | --- |
| Digoxin |  |  |  |
| 1-year wash-out | 1.00 (ref.) | 1.13 (1.01-1.27) | 0.033 |
| 2-year wash-out | 1.00 (ref.) | 1.18 (1.05-1.32) | 0.004 |
| 3-year wash-out | 1.00 (ref.) | 1.23 (1.09-1.38) | <0.001 |
| Amlodipine |  |  |  |
| 1-year wash-out | 1.00 (ref.) | 1.01 (0.90-1.14) | 0.818 |
| 2-year wash-out | 1.00 (ref.) | 0.99 (0.87-1.11) | 0.807 |
| 3-year wash-out | 1.00 (ref.) | 1.02 (0.90-1.15) | 0.742 |
| Amlodipine combination |  |  |  |
| 1-year wash-out | 1.00 (ref.) | 1.18 (1.05-1.32) | 0.005 |
| 2-year wash-out | 1.00 (ref.) | 1.21 (1.08-1.36) | 0.001 |
| 3-year wash-out | 1.00 (ref.) | 1.26 (1.12-1.42) | <0.001 |
| Valsartan |  |  |  |
| 1-year wash-out | 1.00 (ref.) | 0.50 (0.43-0.58) | <0.001 |
| 2-year wash-out | 1.00 (ref.) | 0.49 (0.42-0.57) | <0.001 |
| 3-year wash-out | 1.00 (ref.) | 0.51 (0.44-0.59) | <0.001 |
| Valsartan combination |  |  |  |
| 1-year wash-out | 1.00 (ref.) | 1.20 (1.07-1.35) | 0.002 |
| 2-year wash-out | 1.00 (ref.) | 1.24 (1.11-1.40) | <0.001 |
| 3-year wash-out | 1.00 (ref.) | 1.29 (1.15-1.45) | <0.001 |
| Telmisartan |  |  |  |
| 1-year wash-out | 1.00 (ref.) | 0.95 (0.84-1.07) | 0.385 |
| 2-year wash-out | 1.00 (ref.) | 0.94 (0.83-1.06) | 0.292 |
| 3-year wash-out | 1.00 (ref.) | 0.97 (0.86-1.10) | 0.630 |
| Telmisartan combination |  |  |  |
| 1-year wash-out | 1.00 (ref.) | 0.84 (0.74-0.95) | 0.007 |
| 2-year wash-out | 1.00 (ref.) | 0.86 (0.75-0.97) | 0.017 |
| 3-year wash-out | 1.00 (ref.) | 0.89 (0.78-1.01) | 0.073 |
| Atenolol |  |  |  |
| 1-year wash-out | 1.00 (ref.) | 0.75 (0.66-0.85) | <0.001 |
| 2-year wash-out | 1.00 (ref.) | 0.74 (0.65-0.84) | <0.001 |
| 3-year wash-out | 1.00 (ref.) | 0.77 (0.67-0.88) | <0.001 |
| Furosemide |  |  |  |
| 1-year wash-out | 1.00 (ref.) | 1.00 (0.89-1.13) | 0.960 |
| 2-year wash-out | 1.00 (ref.) | 0.91 (0.80-1.03) | 0.131 |
| 3-year wash-out | 1.00 (ref.) | 0.90 (0.79-1.02) | 0.097 |
| Isosorbide dinitrate |  |  |  |
| 1-year wash-out | 1.00 (ref.) | 0.84 (0.75-0.95) | 0.005 |
| 2-year wash-out | 1.00 (ref.) | 0.90 (0.80-1.01) | 0.075 |
| 3-year wash-out | 1.00 (ref.) | 0.27 (0.22-0.33) | <0.001 |
| Torasemide |  |  |  |
| 1-year wash-out | 1.00 (ref.) | 1.42 (1.26-1.59) | <0.001 |
| 2-year wash-out | 1.00 (ref.) | 1.42 (1.26-1.60) | <0.001 |
| 3-year wash-out | 1.00 (ref.) | 1.15 (1.02-1.30) | 0.024 |

Data are subdistribution hazard ratio calculated using the Fine and Gray’s model after adjustments for age, sex, household income, body mass index, smoking status, moderate-to-vigorous physical activity, and a history of cardiovascular disease. Wash-out period was defined as the period from the start of the follow-up investigation until specified time.
