# Supplementary material for: Identification of potentially effective drugs for metabolic dysfunction-associated steatotic liver disease against liver cirrhosis: In-silico drug repositioning-based retrospective cohort study
Source: PLoS One. 2025 Jun 4;20(6):e0323880. doi: 10.1371/journal.pone.0323880 (PMC12136429; doi:10.1371/journal.pone.0323880)
Supplement: S3 Table — (DOCX) [file pone.0323880.s010.docx]

**S3 Table. Stratified analysis on the risk of liver cirrhosis according to the cumulative days of drug use.**

| **Subgroup** | **<30 days** | **≥30 days** | ***P* value** | ***P* for interaction** |
| --- | --- | --- | --- | --- |
| **Digoxin** |  |  |  |  |
| Age |  |  |  | <0.001 |
| <65 years | 1.00 (ref.) | 1.27 (1.12-1.45) | <0.001 |  |
| ≥65 years | 1.00 (ref.) | 0.85 (0.72-1.01) | 0.070 |  |
| Sex |  |  |  | 0.797 |
| Men | 1.00 (ref.) | 1.23 (1.04-1.45) | 0.018 |  |
| Women | 1.00 (ref.) | 1.07 (0.92-1.26) | 0.374 |  |
| Obesity |  |  |  | <0.001 |
| No | 1.00 (ref.) | 1.48 (1.27-1.73) | <0.001 |  |
| Yes | 1.00 (ref.) | 0.54 (0.45-0.67) | <0.001 |  |
| Charlson comorbidity index |  |  |  | <0.001 |
| 0 | 1.00 (ref.) | NA | NA |  |
| 1 | 1.00 (ref.) | 0.48 (0.39-0.60) | <0.001 |  |
| ≥2 | 1.00 (ref.) | 2.31 (1.98-2.69) | <0.001 |  |
| **Amlodipine** |  |  |  |  |
| Age |  |  |  | 0.001 |
| <65 years | 1.00 (ref.) | 1.06 (0.86-1.31) | 0.589 |  |
| ≥65 years | 1.00 (ref.) | 0.69 (0.53-0.88) | 0.003 |  |
| Sex |  |  |  | <0.001 |
| Men | 1.00 (ref.) | 1.47 (1.20-1.79) | <0.001 |  |
| Women | 1.00 (ref.) | 0.57 (0.43-0.76) | <0.001 |  |
| Obesity |  |  |  | <0.001 |
| No | 1.00 (ref.) | 0.49 (0.36-0.68) | <0.001 |  |
| Yes | 1.00 (ref.) | 1.63 (1.32-2.00) | <0.001 |  |
| Charlson comorbidity index |  |  |  | 0.001 |
| 0 | 1.00 (ref.) | 1.40 (0.83-2.37) | 0.208 |  |
| 1 | 1.00 (ref.) | 0.78 (0.54-1.14) | 0.201 |  |
| ≥2 | 1.00 (ref.) | 0.94 (0.77-1.15) | 0.550 |  |
| **Amlodipine-based combination** |  |  |  |  |
| Age |  |  |  | <0.001 |
| <65 years | 1.00 (ref.) | 0.40 (0.28-0.57) | <0.001 |  |
| ≥65 years | 1.00 (ref.) | 3.36 (2.82-4.00) | <0.001 |  |
| Sex |  |  |  | <0.001 |
| Men | 1.00 (ref.) | 0.58 (0.43-0.78) | <0.001 |  |
| Women | 1.00 (ref.) | 3.33 (2.77-4.02) | <0.001 |  |
| Obesity |  |  |  | 0.002 |
| No | 1.00 (ref.) | 2.10 (1.74-2.54) | <0.001 |  |
| Yes | 1.00 (ref.) | 1.07 (0.83-1.38) | 0.602 |  |
| Charlson comorbidity index |  |  |  | <0.001 |
| 0 | 1.00 (ref.) | 1.22 (0.63-2.35) | 0.552 |  |
| 1 | 1.00 (ref.) | 0.80 (0.54-1.20) | 0.280 |  |
| ≥2 | 1.00 (ref.) | 1.99 (1.66-2.38) | <0.001 |  |
| **Valsartan** |  |  |  |  |
| Age |  |  |  | <0.001 |
| <65 years | 1.00 (ref.) | 4.13 (3.28-5.20) | <0.001 |  |
| ≥65 years | 1.00 (ref.) | 0.80 (0.54-1.17) | 0.247 |  |
| Sex |  |  |  | <0.001 |
| Men | 1.00 (ref.) | 0.14 (0.06-0.33) | <0.001 |  |
| Women | 1.00 (ref.) | 3.72 (3.06-4.51) | <0.001 |  |
| Obesity |  |  |  | 0.029 |
| No | 1.00 (ref.) | 2.45 (1.95-3.09) | <0.001 |  |
| Yes | 1.00 (ref.) | 1.00 (0.68-1.48) | 0.993 |  |
| Charlson comorbidity index |  |  |  | <0.001 |
| 0 | 1.00 (ref.) | 1.24 (0.31-4.93) | 0.765 |  |
| 1 | 1.00 (ref.) | 5.15 (4.15-6.39) | <0.001 |  |
| ≥2 | 1.00 (ref.) | 0.63 (0.44-0.91) | 0.014 |  |
| **Valsartan-based combination** |  |  |  |  |
| Age |  |  |  | <0.001 |
| <65years | 1.00 (ref.) | 0.37 (0.23-0.61) | <0.001 |  |
| ≥65years | 1.00 (ref.) | 3.24 (2.71-3.87) | <0.001 |  |
| Sex |  |  |  | <0.001 |
| Men | 1.00 (ref.) | 0.39 (0.25-0.61) | <0.001 |  |
| Women | 1.00 (ref.) | 3.40 (2.83-4.09) | <0.001 |  |
| Obesity |  |  |  | 0.001 |
| No | 1.00 (ref.) | 2.47 (2.02-3.02) | <0.001 |  |
| Yes | 1.00 (ref.) | 1.17 (0.88-1.57) | 0.282 |  |
| Charlson comorbidity index |  |  |  | <0.001 |
| 0 | 1.00 (ref.) | 0.79 (0.34-1.86) | 0.588 |  |
| 1 | 1.00 (ref.) | 0.56 (0.31-1.02) | 0.056 |  |
| ≥2 | 1.00 (ref.) | 2.38 (1.98-2.85) | <0.001 |  |
| **Telmisartan** |  |  |  |  |
| Age |  |  |  | 0.945 |
| <65 years | 1.00 (ref.) | 0.52 (0.25-1.10) | 0.088 |  |
| ≥65 years | 1.00 (ref.) | 0.49 (0.26-0.95) | 0.036 |  |
| Sex |  |  |  | 0.929 |
| Men | 1.00 (ref.) | 0.44 (0.21-0.93) | 0.032 |  |
| Women | 1.00 (ref.) | 0.62 (0.32-1.19) | 0.146 |  |
| Obesity |  |  |  | 0.025 |
| No | 1.00 (ref.) | 0.25 (0.09-0.65) | 0.005 |  |
| Yes | 1.00 (ref.) | 0.52 (0.28-0.98) | 0.042 |  |
| Charlson comorbidity index |  |  |  | 0.471 |
| 0 | 1.00 (ref.) | 0.68 (0.10-4.81) | 0.702 |  |
| 1 | 1.00 (ref.) | 0.97 (0.36-2.62) | 0.946 |  |
| ≥2 | 1.00 (ref.) | 0.45 (0.25-0.81) | 0.008 |  |
| **Telmisartan-based combination** |  |  |  |  |
| Age |  |  |  | 0.129 |
| <65 years | 1.00 (ref.) | 3.31 (2.71-4.03) | <0.001 |  |
| ≥65 years | 1.00 (ref.) | 3.08 (2.58-3.69) | <0.001 |  |
| Sex |  |  |  | 0.018 |
| Men | 1.00 (ref.) | 2.00 (1.61-2.49) | <0.001 |  |
| Women | 1.00 (ref.) | 3.77 (3.13-4.54) | <0.001 |  |
| Obesity |  |  |  | 0.408 |
| No | 1.00 (ref.) | 3.63 (3.02-4.37) | <0.001 |  |
| Yes | 1.00 (ref.) | 2.80 (2.27-3.44) | <0.001 |  |
| Charlson comorbidity index |  |  |  | <0.001 |
| 0 | 1.00 (ref.) | 0.58 (0.18-1.85) | 0.354 |  |
| 1 | 1.00 (ref.) | 0.49 (0.23-1.01) | 0.053 |  |
| ≥2 | 1.00 (ref.) | 4.82 (4.04-5.74) | <0.001 |  |
| **Atenolol** |  |  |  |  |
| Age |  |  |  | 0.585 |
| <65 years | 1.00 (ref.) | 0.28 (0.11-0.69) | 0.006 |  |
| ≥65 years | 1.00 (ref.) | 0.40 (0.23-0.69) | 0.001 |  |
| Sex |  |  |  | 0.952 |
| Men | 1.00 (ref.) | 0.38 (0.20-0.71) | 0.003 |  |
| Women | 1.00 (ref.) | 0.40 (0.21-0.77) | 0.006 |  |
| Obesity |  |  |  | 0.208 |
| No | 1.00 (ref.) | 0.24 (0.12-0.52) | <0.001 |  |
| Yes | 1.00 (ref.) | 0.48(0.27-0.85) | 0.012 |  |
| Charlson comorbidity index |  |  |  | 0.026 |
| 0 | 1.00 (ref.) | 0.65 (0.20-2.09) | 0.471 |  |
| 1 | 1.00 (ref.) | 0.63 (0.32-1.26) | 0.188 |  |
| ≥2 | 1.00 (ref.) | 0.21 (0.10-0.43) | <0.001 |  |
| **Furosemide** |  |  |  |  |
| Age |  |  |  | <0.001 |
| <65 years | 1.00 (ref.) | 1.44 (1.22-1.69) | <0.001 |  |
| ≥65 years | 1.00 (ref.) | 2.19 (1.83-2.64) | <0.001 |  |
| Sex |  |  |  | 0.007 |
| Men | 1.00 (ref.) | 2.12 (1.80-2.50) | <0.001 |  |
| Women | 1.00 (ref.) | 1.67 (1.38-2.03) | <0.001 |  |
| Obesity |  |  |  | <0.001 |
| No | 1.00 (ref.) | 1.28 (1.06-1.53) | 0.010 |  |
| Yes | 1.00 (ref.) | 2.34 (1.94-2.81) | <0.001 |  |
| Charlson comorbidity index |  |  |  | <0.001 |
| 0 | 1.00 (ref.) | NA | NA |  |
| 1 | 1.00 (ref.) | 0.08 (0.03-0.24) | <0.001 |  |
| ≥2 | 1.00 (ref.) | 4.07 (3.50-4.72) | <0.001 |  |
| **Isosorbide dinitrate** |  |  |  |  |
| Age |  |  |  | <0.001 |
| <65 years | 1.00 (ref.) | 6.27 (4.89-8.02) | <0.001 |  |
| ≥65 years | 1.00 (ref.) | 0.30 (0.10-0.94) | 0.038 |  |
| Sex |  |  |  | <0.001 |
| Men | 1.00 (ref.) | 4.56 (3.68-5.64) | <0.001 |  |
| Women | 1.00 (ref.) | 0.15 (0.02-1.03) | 0.054 |  |
| Obesity |  |  |  | <0.001 |
| No | 1.00 (ref.) | 0.11 (0.02-0.76) | 0.025 |  |
| Yes | 1.00 (ref.) | 5.45 (4.39-6.77) | <0.001 |  |
| Charlson comorbidity index |  |  |  | <0.001 |
| 0 | 1.00 (ref.) | NA | NA |  |
| 1 | 1.00 (ref.) | 0.48 (0.15-1.56) | 0.222 |  |
| ≥2 | 1.00 (ref.) | 4.63 (3.63-5.92) | <0.001 |  |
| **Torasemide** |  |  |  |  |
| Age |  |  |  | 0.002 |
| <65 years | 1.00 (ref.) | 4.16 (3.37-5.13) | <0.001 |  |
| ≥65 years | 1.00 (ref.) | 2.79 (2.21-3.53) | <0.001 |  |
| Sex |  |  |  | <0.001 |
| Men | 1.00 (ref.) | 1.59 (1.26-2.01) | <0.001 |  |
| Women | 1.00 (ref.) | 8.16 (6.81-9.77) | <0.001 |  |
| Obesity |  |  |  | 0.914 |
| No | 1.00 (ref.) | 3.73 (3.09-4.50) | <0.001 |  |
| Yes | 1.00 (ref.) | 2.53 (1.99-3.22) | <0.001 |  |
| Charlson comorbidity index |  |  |  | <0.001 |
| 0 | 1.00 (ref.) | NA | NA |  |
| 1 | 1.00 (ref.) | 3.82 (3.07-4.76) | <0.001 |  |
| ≥2 | 1.00 (ref.) | 3.92 (3.22-4.77) | <0.001 |  |

Data are subdistribution hazard ratio calculated using the Fine and Gray’s model after adjustments for age, sex, household income, body mass index, smoking status, moderate-to-vigorous physical activity, and a history of cardiovascular disease.
