# Supplementary material for: Identification of potentially effective drugs for metabolic dysfunction-associated steatotic liver disease against liver cirrhosis: In-silico drug repositioning-based retrospective cohort study
Source: PLoS One. 2025 Jun 4;20(6):e0323880. doi: 10.1371/journal.pone.0323880 (PMC12136429; doi:10.1371/journal.pone.0323880)
Supplement: Attachment — Submitted filename: PONE-D-24-48206 - Reviewer comments.pdf [file pone.0323880.s011.pdf]

**REVIEWER COMMENTS: In Silico Drug Repositioning-based Identification of Potentially Effective Drugs for 2 Metabolic Dysfunction-Associated Steatotic Liver Disease against Liver Cirrhosis**

Based on review of clinical health screening data, Authors propose a signature-based in silico approach to identify specific drugs that could be used to mitigate a risk of liver cirrhosis in MASLD patients. To the best of my knowledge, the study presents original research that have not been published elsewhere. Despite the evidence provided, the manuscript has several areas that require addressing.

It is unclear how the identified 415 differentially expressed genes impacted the identification of digoxin – can the authors please provide more specific information and some additional clarity on that finding? No information is provided on the DEGs that were up/down-regulated, the pathways they impact, or any commonality/relatedness between them.

The initial identification of digoxin appears somewhat random and fortuitous, as it was the only drug with a matching Korean ATC code. The basis of additional related-drug selections followed on from that initial 'identification'. Can the authors please provide some clarity on whether any hypotheses were generated before assessing these databases on the potential drugs and/or their related mechanism of action that they expected may be implicated?

Please provide specifics on how "a meticulous validation study to assess the prospective efficacy of Digoxin using extensive patient data from the NHIS" will be performed? Also, the data provided herein indicated that use of Digoxin is associated with a higher risk of liver cirrhosis, not lower, therefore the suggestion to evaluate its 'prospective efficacy' seems unwarranted.

In essence, this paper is hypothesis generating, based on currently available database information, without any formal validation performed, which the authors contend that this will be provided in a follow up study. While the association data provided on the potential impact of atenolol, isosorbide dinitrate and valsartan on lowered risk of liver cirrhosis are of interest, it is unclear based on the information provided in the manuscript how the signature-based methodology played any part in supporting these findings. Additional clarity on the exact methods used to support this are crucial to supporting the overall potential of such in silico methodologies in identifying potentially effective drugs to MASLD.
